# Extrahepatic Manifestations of Chronic Hepatitis C Virus (HCV) Infection

**DOI:** 10.7759/cureus.57343

**Published:** 2024-03-31

**Authors:** Bella Garg, Amirmohsen Arbabi, Purnell A Kirkland

**Affiliations:** 1 Internal Medicine/Rheumatology, Centinela Hospital, Los Angeles, USA; 2 Internal Medicine, Centinela Hospital, Los Angeles, USA; 3 Rheumatology, Centinela Hospital, Los Angeles, USA

**Keywords:** hepatitis c virus (hcv), iv drug abusers, end-stage kidney disease, essential mixed cryoglobulinemia, extrahepatic manifestations

## Abstract

Hepatitis C virus (HCV) is a well-recognized, major cause of various liver-related conditions such as chronic hepatitis, liver cirrhosis, and hepatocellular carcinoma. Apart from liver disease, chronic HCV infection is also associated with several extrahepatic manifestations that can lead to significant morbidity and mortality. These extrahepatic manifestations include essential mixed cryoglobulinemia (EMC), lymphomas, porphyria cutanea tarda, lichen planus, necrolytic acral erythema, glomerulonephritis, subclinical autoantibody formation, immune thrombocytopenia, thyroid disease, Sjögren's disease/sicca symptoms, diabetes mellitus, ocular diseases, musculoskeletal disorders, cardiovascular diseases, neurocognitive dysfunction, and leukocytoclastic vasculitis. We are presenting a case of chronic HCV infection linked to the extrahepatic manifestations of the disease which can be directly related to HCV or indirectly related to EMC from HCV. An awareness and knowledge of these extrahepatic manifestations will highlight the importance of recognizing the symptoms for an early diagnosis and effective anti-viral treatment in order to improve or resolve the long-term complications of chronic HCV infection.

## Introduction

Hepatitis C is a viral disease that leads to inflammation of the liver. It can cause both acute and chronic hepatitis, ranging from a mild illness to a serious, long-term illness including liver cirrhosis and hepatocellular carcinoma (HCC). Worldwide, an estimated 58 million people have chronic hepatitis C virus (HCV) infection, with about 1.5 million new HCV infections annually [1]. The World Health Organization (WHO) estimated that in 2019, approximately 290,000 people died from hepatitis C, mostly from liver cirrhosis and HCC [1].

Chronic HCV infection is associated with a variety of extrahepatic manifestations as a systemic disease. These manifestations are common and include hematologic disorders such as essential mixed cryoglobulinemia (EMC), lymphoma, and monoclonal gammopathies; autoimmune disorders such as thyroiditis, asymptomatic autoantibodies, Sjögren's disease/sicca symptoms, autoimmune hepatitis, immune thrombocytopenia, and autoimmune hemolytic anemia; renal diseases such as membranoproliferative glomerulonephritis (GN) and membranous GN; dermatologic conditions such as lichen planus, porphyria cutanea tarda, necrolytic acral erythema, and leukocytoclastic vasculitis; diabetes mellitus; neurologic and neuropsychiatric diseases; cardiac and cardiovascular diseases; musculoskeletal conditions; and ocular diseases [2,3]. Chronic HCV infection is an economic burden, and the extrahepatic manifestations can influence the morbidity, health-related quality of life, and mortality of these patients [4].

This case report aims to provide awareness regarding the importance of early detection of HCV infection within high-risk populations so that appropriate curative treatment can prevent serious future health complications such as EMC and its consequential hemodialysis (HD)-dependent end-stage kidney disease (ESKD).

## Case presentation

This case highlights a 66-year-old male with a history of incarceration and intravenous (IV) heroin use 25 years ago who presented to the emergency department (ED) with a chief complaint of persistent generalized weakness and fatigue for the past six months. He reports that he was diagnosed with HCV infection 20 years ago for which he was treated with IV interferon; however, the course of the medication was not completed due to intolerable adverse effects that included weakness, fatigue, gastrointestinal symptoms, and severe neutropenia. Initial vital signs on admission revealed a temperature of 37°C, a blood pressure of 125/80 mmHg, a pulse rate of 95/minute, and a respiratory rate of 15/minute with an oxygen saturation of 99% on room air. Additionally, the patient complained of recurrent hemoptysis, shortness of breath, and cough. In the last several months, his kidney function also rapidly deteriorated, which finally required him to begin scheduled HD. He also experienced progressively worsening episodes of musculoskeletal symptoms including arthralgia in multiple joints, severe lower extremity myalgia, and numbness and tingling in his extremities. Furthermore, he reports bilateral non-healing ulcers on his both knees following a mechanical fall. The patient endorses that the abrasions developed after a mechanical fall down the stairs last month due to a sudden onset of fatigue and muscle weakness. He mentions that the abrasions have never healed even after receiving a course of oral antibiotics for 10 days following the fall.

**Figure 1:**
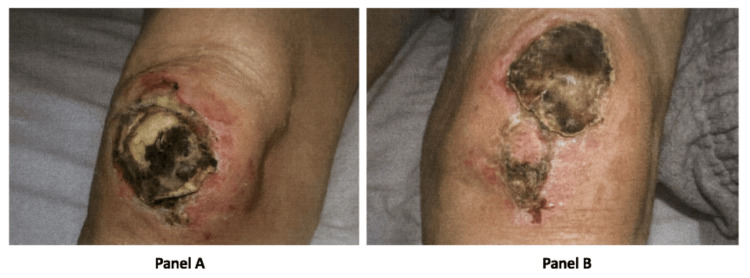
10x10 cm non-painful, pruritic black eschars with central ulcerations on both knees, panel A (right knee) and panel B (left knee).

Significant findings on physical examination included decreased bibasilar breath sounds on auscultation, 10x10 cm non-painful, pruritic black eschars with central ulcerations on both knees (Figure 1), and +1 pitting edema bilaterally on the lower extremities. Upon closer inspection, there was also joint tenderness bilaterally in both his hands and knees, as well as muscle tenderness localized mainly in his lower extremities.

The initial laboratory evaluation displayed the presence of normocytic anemia with a hemoglobin of 8.5 g/dL. The kidney function tests presented with a creatinine level of 8.7 mg/dL, a blood urea nitrogen of 103 mg/dL, a glomerular filtration rate of 7 mL/min, and a bicarbonate level of 14 mEq/L. The corrected calcium was low at 6.9 mg/dL with a high phosphorus level of 10.2 mg/dL, a high parathyroid hormone level of 527 pg/mL, and a low vitamin D level of 30.2 ng/mL. The urinalysis (UA) was not obtainable as the patient was anuric at presentation. His liver function tests showed an aspartate aminotransferase of 3419 IU/L, an alanine aminotransferase of 760 IU/L, and an alkaline phosphatase of 257 IU/L. The inflammatory markers revealed an elevated procalcitonin level of 33.43 ng/mL and an elevated erythrocyte sedimentation rate of 88 mm/hr.

**Figure 2:**
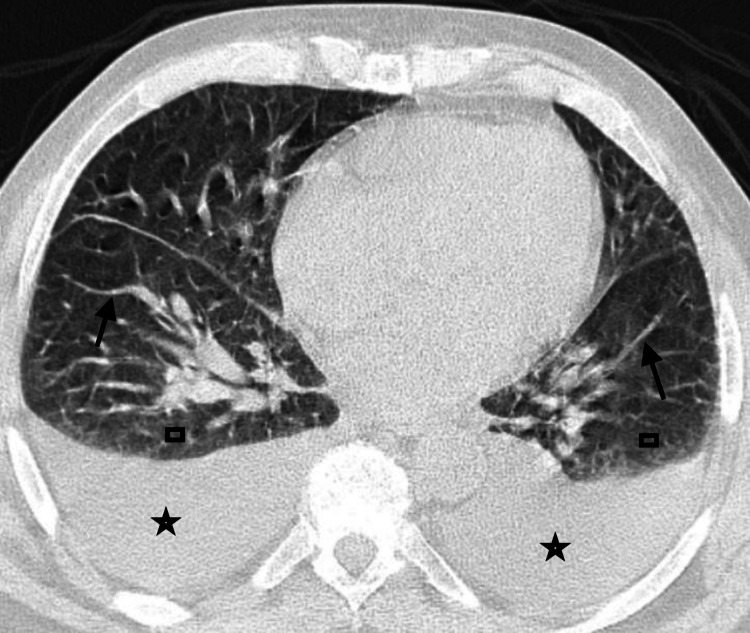
The chest CT scan shows bilateral pleural effusions (stars) with septal thickening (arrows) and patchy infiltrates (rectangles). CT: computed tomography

Further assessments included blood and wound cultures, and the patient was started empirically on vancomycin and piperacillin-tazobactam IV in the ED. He was admitted to the medicine service for further evaluation and management. Over the course of 10 days, multiple subspecialties were consulted including nephrology, pulmonology, rheumatology, infectious diseases (ID), and wound care. The nephrology service decided not to perform a kidney biopsy due to the patient's low hemoglobin level and ESKD; therefore, they directly proceeded with HD. The ID service decided to continue the empiric antibiotic therapy, and the wound care provided treatment for the patient's abrasions daily. The pulmonology service requested a computed tomography (CT) scan, which showed bilateral pleural effusions with septal thickening and patchy infiltrates (Figure 2).

**Figure 3:**
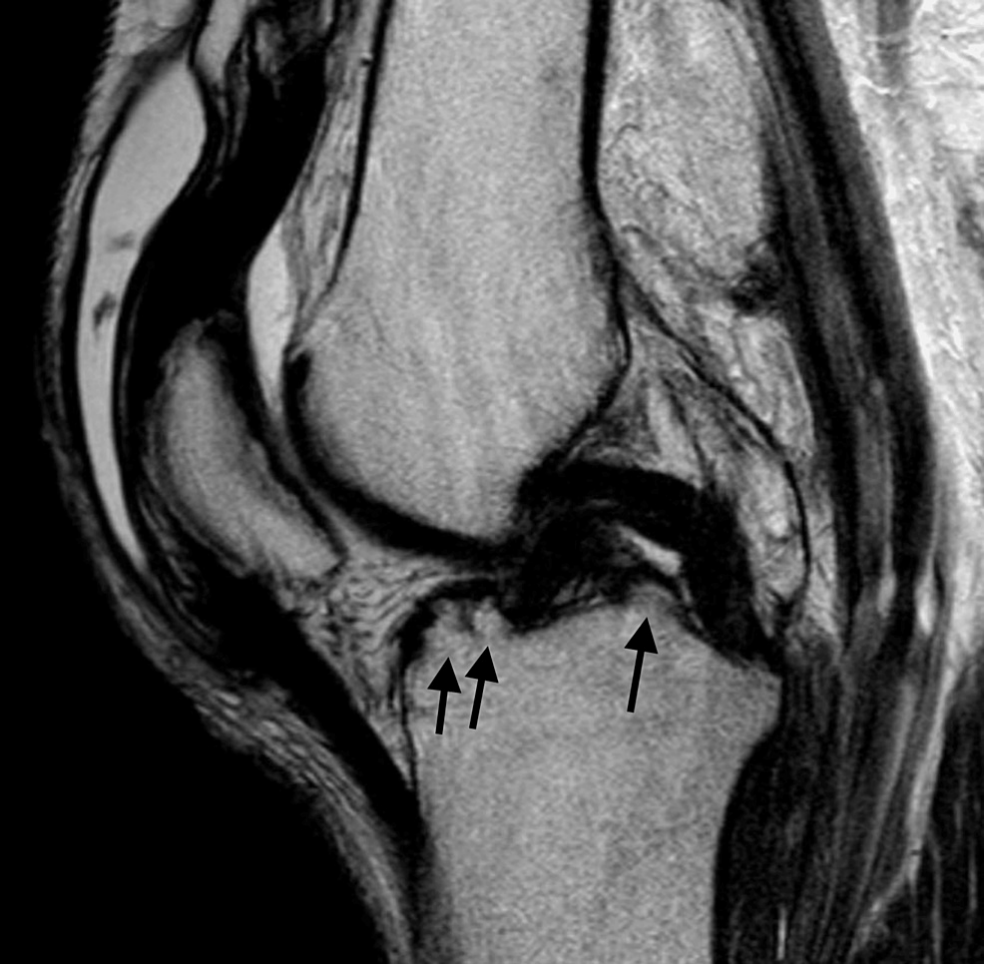
The MRI of the bilateral knees shows pre-patellar soft tissue swelling and possible intra-articular loose bodies (arrows). MRI: magnetic resonance imaging

The hepatitis panel showed elevated HCV antibody (>11.0 index value) with a non-reactive human immunodeficiency virus fourth-generation test. Additionally, it was noted that the aldolase level was elevated at 13.7 U/L and the creatine kinase level was significantly elevated at 242,000 IU/L. Other pertinent labs revealed an elevated level of uric acid at 9.6 mg/dL and potassium at 6.6 mEq/L. The rheumatoid factor was elevated at 46.2 IU/mL, the immune complex C1Q binding was elevated at 18.2 ug Eq/mL, and the complement 3 and 4 levels were normal. Magnetic resonance imaging (MRI) was obtained for both his knees and was negative for any evidence of arthritis/osteomyelitis; however, there was evidence of pre-patellar soft tissue swelling and possible intra-articular loose bodies (Figure 3).

The rheumatology service requested more lab tests to investigate for other immunologic diseases such as HCV-related EMC, anti-neutrophilic cytoplasmic antibody-associated vasculitides, rheumatoid arthritis, polymyositis, and dermatomyositis. Based on these findings, the rheumatology service was highly suspicious of extrahepatic manifestations of chronic hepatitis C infection and HCV-related EMC. Subsequently, the patient was placed on high-dose prednisone plus rituximab. Once final blood and wound cultures were available, they were negative for any microbe growth, and thus, IV antibiotic therapy was discontinued. On the 10th day of the patient's hospitalization, he was medically stable for discharge. The patient was informed to schedule a follow-up appointment as an outpatient with his primary care physician and rheumatologist in one week. He was also advised to follow up with the ID clinic to begin anti-viral treatment for his chronic HCV infection.

## Discussion

HCV infection predominantly affects the liver leading to conditions such as acute and chronic hepatitis, cirrhosis, portal hypertension, hepatic encephalopathy, and HCC [5]. However, extrahepatic manifestations of chronic HCV infection are also common and include hematologic diseases such as EMC and lymphoma, autoimmune disorders such as thyroiditis, renal diseases, and dermatologic conditions such as lichen planus and porphyria cutanea tarda [6].

Chronic HCV infection can also manifest primary and secondary complications in various organs. For example, negative effects on the lungs can show exacerbation of the existing chronic obstructive pulmonary disease and asthma or, less frequently, interstitial lung diseases such as pulmonary fibrosis [7]. Subsequently, EMC can also exhibit subclinical pulmonary presentations such as small airway disease and impairment of gas exchange resulting in symptoms such as dyspnea, cough, and pleurisy [8]. Occasionally, EMC can also cause organizing pneumonia, alveolar hemorrhage, and pulmonary vasculitis [9]. Our patient's lung involvement can be either directly explained by chronic HCV infection itself or indirectly observed in HCV-related EMC.

Renal complications linked to chronic HCV infection include glomerular diseases most commonly, membranoproliferative GN which is usually associated with EMC, and, less frequently, membranous GN [10,11]. As mentioned earlier, a kidney biopsy could not be done due to the anemia and ESKD, so unfortunately, we are unable to verify whether his kidney disease was directly related to HCV infection or indirectly related to EMC. The Kidney Disease Improving Global Outcomes (KDIGO) clinical practice guidelines recommend screening for kidney disease at the time of HCV infection diagnosis and then annually thereafter with follow-up labs including a UA and serum creatinine [12].

Musculoskeletal disorders associated with HCV infection include decreased bone mineral density, fractures, osteosclerosis, arthralgia, arthritis, and myalgia [13-15]. Between 2% and 20% of patients with HCV infection are reported to have arthritis, two-thirds of which was rheumatoid-like and the remaining exhibiting oligoarthritis. Myopathy and myositis are also reported to be associated with HCV infection [16,17]. Like in our case, the possibility of EMC should also be considered in HCV-infected patients who presented with arthralgias or myalgias [18,19]. Arthralgias are reported in nearly 70% of patients with EMC, often in type III EMC, especially affecting the proximal phalangeal and metacarpophalangeal joints, knees, and ankles [18,19].

A variety of dermatologic diseases are associated with HCV infection such as porphyria cutanea tarda, lichen planus, necrolytic acral erythema, and leukocytoclastic vasculitis [20]. Leukocytoclastic vasculitis can also occur in conjunction with EMC presenting clinically with lower extremity palpable purpura and petechiae [21]. The vasculitis can also result in skin ischemic necrosis and ulceration. Similar vasculitic changes can affect the lower extremity peripheral nerves manifesting as a peripheral neuropathy that is typically asymmetric (mononeuritis multiplex) [22].

## Conclusions

Recognizing the extrahepatic manifestations associated with HCV and understanding the significance of its early detection and treatment in individuals at high risk (incarceration and IV drug user) are crucial to avoid major health issues associated with chronic HCV infection such as cirrhosis, liver transplantation, ESKD, and dependence on HD. Finally, antiviral treatment, glucocorticoids, and/or immunosuppressors may improve the wide variety of symptoms associated with chronic HCV infection and should be considered in such patients.
